# The Influence of Wind Musical Instruments on the Orofacial System

**DOI:** 10.3390/diagnostics14202342

**Published:** 2024-10-21

**Authors:** Raquel Laparra Hernández, Santiago Arias-Luxán, Salvatore Sauro, Alicia Lanuza

**Affiliations:** 1 Department of Dentistry, Institute of Biomedical Sciences, Cardenal Herrera-CEU University, CEU Universities, 46115 Valencia, Spain; santiago.arias@uchceu.es (S.A.-L.); salvatore.sauro@uchceu.es (S.S.); 2 Department of Dental Medicine, Faculty of Medicine and Dentistry, University of Valencia, 46010 Valencia, Spain; alicia.lanuza@uv.es

**Keywords:** malocclusion, wind instruments, music practice, orofacial system

## Abstract

Objectives: The main objective of this study was to assess whether there are differences in the muscular and occlusal levels between wind players and the general population, in addition to assessing the different repercussions depending on the embouchure type. Material and Methods: Two cohorts were chosen: one of them comprised intermediate students related to the practice of a wind instrument (n = 39), and the other one, harmonized with the former, comprised secondary students that served as a control group (n = 19). Overjet, overbite, presence of lip abrasion and/or erosion, Angle’s molar class, Little’s irregularity index, dental abrasion, presence of labial herpes, presence of tooth sensitivity, and presence of articular noises muscle pain were evaluated. Results: Significant statistical differences between the groups were found regarding overjet, overcrowding of teeth, lower lip erosion, and dental abrasion, which was higher in musicians. Lastly, within the group of musicians, it was proven that tooth sensitivity was higher in the group of brass musicians. Conclusions: Wind instrument practice may be a risk factor for developing some orofacial involvement, and there are no differences among the various existing mouthpieces.

## 1. Introduction

Recently, the number of children who start playing musical instruments has been increasing—and is continuing to increase—although there are few studies that prove the influence that instruments might have on the orofacial system and/or vice versa. Among the different musical instruments, those which might have any sort of influence are wind instruments, among which we can distinguish between woodwind instruments and brass instruments [1]. Moreover, woodwind instruments can be classified according to their mouthpiece, that is, single-reed mouthpiece, double-reed mouthpiece, and hole mouthpiece [1].

In a survey conducted among instrument teachers in a previous study [2], 60% of them believed that playing a wind instrument might affect the mouth, while 91.6% of them thought there was a difference regarding the adaptation between a child who had had good teething and another one who had not. That difference caused variations, from a greater to lesser degree, in the sound, position of the mouthpiece, marcato, tuning, and pain felt in some parts of the oral cavity when playing the instrument [2].

Therefore, it is important to carry out studies that assess the impact of the instruments on patient malocclusion.

**Figure 1:**
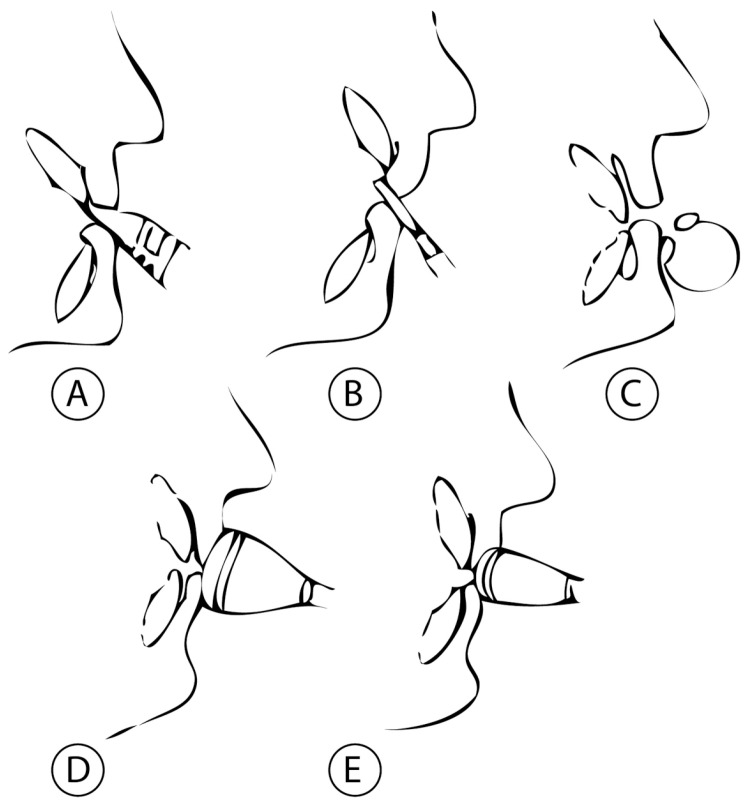
Summary of the classification of the different mouthpieces: (**A**) single-reed mouthpiece; (**B**) double-reed mouthpiece; (**C**) hole or aperture mouthpiece; (**D**) cup-shaped mouthpiece; (**E**) cup-shaped mouthpiece.

In fact, there are some recommendations for each type of mouthpiece according to Angle’s classifications of malocclusion [1,3] (Figure 1):

Angle Class II. Double-reed woodwind and brass instruments are indicated in Class II division 1, while single-reed woodwind instruments are contraindicated.

Angle Class III. Single-reed woodwind instruments are indicated for Class III. However, with this kind of malocclusion, it will be difficult to play double-reed woodwind, flute, and brass instruments.

In particular, the morphological variability, together with the presence of malocclusion, might affect the instrument’s angle and position [4], the tuning, and/or the sound produced by the instrument, among others [4,5]. However, for better or worse, any foreign body that comes between the mouth and the instrument, such as the presence of orthodontic appliances, might also affect them.

Suppose that orthodontic treatment starts after first contact with the instrument, as usually happens. In that case, that treatment becomes an obstacle to keeping the already-learned position of the mouth. This means that the person needs to learn how to position their mouth again, and that process takes from 1 to 3 months. This particularly affects brass musicians [6]. Therefore, music teachers are becoming aware of the importance of taking a prior thorough orthodontic examination of children who are going to play a wind instrument [7].

Besides good mouth morphology, it is important to maintain good oral health. It has been observed that the oral hygiene of musicians is good, or at least, it is not that much worse than that of the public. However, musicians do show poor periodontal health [8]. As a result, it has been suggested that playing wind instruments is a periodontal risk factor [9]. The periodontal problems that wind musicians show are due to three reasons: continuous pressure, even if intermittent, on teeth, accompanied by a decrease in capillary circulation in the bone; the strength in the jaw elevator muscles increases the lingual pressure on the anterior maxillary teeth, which are pressed against a hard inclined plane; the production of a higher quantity of saliva than that produced by someone who does not play an instrument, together with greater build-up of calculus [10].

Furthermore, it is necessary to highlight that having good oral and functional health is not the only important factor in performing music. It is also important to bear in mind that a disorder in the orofacial system could also alter a person’s music performance: wind instruments are a foreign element in the mouth that might cause, in the short or long term, mouth disorders such as a change in their overjet [10,11,12,13] and/or in their overbite [12], or higher overcrowding of teeth [14], among others.

The tooth requires a strength level that exceeds the minimum threshold in size and duration to be “moved”, although there is little evidence regarding the optimal force [15]. The force exerted when playing wind instruments is higher than the minimum necessary force (35–60 gr.) to move a tooth (inclination, rotation, and extrusion), which is 500 gr for brass instruments, 270 gr for double/single-reed woodwind instruments, and 211 gr for head joint instruments [16]. These forces also vary depending on whether the player produces high notes, which require greater tension and less opening of the lips, or brass, which is the opposite [10].

Therefore, tooth movement while playing a musical instrument depends on the type of mouthpiece, how many hours the instrument is played, the position of the teeth, and on the force created by the tongue and the oral muscles during the musical performance. It should be considered that, for this tooth movement to be caused, those strengths should be applied for more than 6 h a day [17]. Several studies conducted with animals have shown that if forces are applied for 8 h a day, there is indeed tooth movement [13,18]. Musicians do not usually play for as many hours a day. The duration is variable, and it mainly depends on the instrumentalist. It might vary from 30–60 min—concerts and/or classes not included—to 5 h a day [13,19]. However, it is possible that due to more intense rehearsals, that duration may be increased at the end of the day.

It should also be highlighted that there are different types of forces, that is, continuous, intermittent, and interrupted forces. The forces that affect the most and more quickly are continuous forces [18], which is, however, not the case for wind instruments, as the forces involved in wind instruments are more like intermittent forces. Even so, Oppenheim [20] suggested that intermittent forces are less detrimental to tooth movement, as they provide relaxation periods during which periodontal tissues can regenerate. However, other authors suggest the non-continuous forces to be the most convenient, as they stop root resorption and allow for the healing process to occur [21,22,23].

Therefore, instrumentalists show higher overcrowding of teeth [14], although the presence of tooth movement could also be studied. It has also been observed that overjet tends to increase for single-reed woodwind instrumentalists if there is incisor protrusion; in the case of double-reed woodwind and brass instrumentalists [10,11,12,13], it tends to decrease if there is retrusion. On the contrary, overbite significantly increases for double-reed and head joint woodwind instrumentalists, and a growing trend is observed for single-reed woodwind instrumentalists [12].

Similarly, one often finds erosions and/or lip ulcerations [24,25,26] in wind instrumentalists. They are also vulnerable to suffering from herpes lesions in the lower lip [27]. Additionally, when playing a musical instrument, a strain in the musculoskeletal system of the face is produced, and therefore several disorders, such as articular noises [27,28,29] and muscular pain [7,29,30], are noticeable.

Lastly, it is worth stressing that instrumental practice is a parafunctional activity, in which upper incisors exert force on the mouthpiece in order to hold it. Due to this pressure and to the constant friction of incisors on the mouthpiece, loss of surface and substance in the dental crown’s hard structures [14] and/or tooth sensitivity [12,25,26,31] can be seen. 

In 2014 [2], we conducted a cross-sectional study in which some students of the Conservatory of Music Joaquín Rodrigo and some senior students of odontology at the University of Valencia were compared. It could be observed that there were indeed differences between both groups. Musicians showed poorer hygiene, higher overcrowding of teeth, and higher lip erosion. Moreover, it was more painful for them when their orbicularis and buccinator muscles were touched. 

Although some significant differences were found in certain variables, some limitations led to the proposal of a new longitudinal study focusing on wind instrumentalists in their degree year. It could be thus evaluated if a real cause–effect relationship exists between the studied factors and instrumental practice. It could also be analyzed by its evolution over time.

The main objective of this study was to assess whether there are differences in muscular and occlusal levels between wind players and the general population, in addition to assessing the different repercussions depending on the type of embouchure. On the other hand, it was also observed whether oral hygiene in musicians was equal to that of the general population or worse.

## 2. Materials and Methods

### 2.1. Study Population

This study was a longitudinal cohort study where a group design was created that matched sex and age; thus, a control group was selected from high school students for this purpose. 

The initial study population comprised 39 musical education students (1º professional grade) in the different Conservatories of Valencia who were linked with the practice of wind instruments (experimental group), and 19 non-musicians (secondary school students of the IES Camp de Turia) as a control group.

This choice of sample within the experimental group could not be achieved following well-adjusted, random, and homogeneous criteria by instrument family given the low number of musical education students studying in the conservatories. For this reason, any student who was in the 1st year of professional education could participate in this study. Due to this situation, to harmonize the sample, we tried to find control subjects of the same age and sex; therefore, it was decided to use secondary school students.

In each of the conservatories, all students of 1º professional grade were invited to participate in this study. Every musician was able to participate if they were willing to do so and met the inclusion criteria (Table 1).

It was decided to choose first-year professional degree students because it is the time during which students begin their formal studies and dedicate an average of 8–9 h per week between study hours and master classes. Previously, in the previous two years when they first picked up the instrument, they only played 1–2 h a week. Both study groups were followed over 2 years.

Informed consent was obtained from each one of the individuals taking part in this study, together with authorization provided by the Human Research Ethics Committee of the University of Valencia (H1261341632944).

### 2.2. Dental Exploration Instrumentation

The oral cavity was explored using a mirror, a probe, a periodontal probe, a millimeter ruler, and an ethyl chloride spray. A single examiner conducted the exploration.

### 2.3. Methodology

Three oral explorations with one year of separation among them were conducted (T0, T1, and T2), with T0 being the first of the explorations

#### 2.3.1. Evaluation of Oral Hygiene

To evaluate the oral hygiene, the following variables were registered in each session: the Silness–Löe index (SLI) (Equation (1), Table 2 and Table 3) and community periodontal index of treatment needs (CPITN) (Table 4) with the help of sextants.

Equation (1) Silness–Löe index

(1) $$Silness\;and\;Löe\;Index=\frac{Sum\;of\;scores\;obtaine\;(degrees)}{number\;of\;teeth}$$

#### 2.3.2. Evaluation of Muscular and Occlusal Levels

To evaluate this section, different variables are analyzed: overjet (in millimeters), overbite (in thirds), presence of lip abrasion and/or erosion, Angle’s molar class, Little’s irregularity index, dental abrasion (Table 5), presence of labial herpes, presence of tooth sensitivity (ethyl chloride in pellet form was used to press on the tooth surface), and presence of articular noises and muscle pain when touching the muscles of the jaw, internal pterygoid muscles, orbicularis and buccinators muscles, temporal muscle, sternocleidomastoid muscle, and zygomatic muscle.

Additionally, the intraobserver error was analyzed, in which the examiner repeated the measurements of a series of parameters in two of the evaluation sessions on a total of ten individuals.

### 2.4. Statistical Analysis

The data were analyzed using the SPSS 15.0 program. The significance level used in the analysis was 5% (α = 0.05), a power of 0.97 for the intrasubject effect over time, and 0.57 for the intersubject effect (difference between groups). The general strategy consisted of:

-Descriptive statistics, sample homogeneity, and interobserver error:

Median, standard deviation, minimum, maximum, and median for the continuous variables, and the absolute and relative frequencies for the categorical variables.

Samples’ homogeneity for the first exploration: The chi2 test was used, together with Fisher’s exact test (in 2 × 2 tables with expected low frequencies in cells) and the non-parametric Mann–Whitney test.

Interobserver error: Dahlberg’s d calculation for continuous parameters and kappa index calculation following categorical parameters were used.

-Instrumental practice assessment: Generalized estimating equation (EEG) models were estimated. However, it was not possible to use EEG for some response variables showing zero prevalence in some measure times. Therefore, Fisher’s test was applied (non-parametric test), to compare if the temporal evolution was similar for both groups.

-Assessment of the type of mouthpiece: The Kruskal–Wallis test was used.

-A comparative assessment regarding oral health and state: Generalized estimating equation (EEG) models were used.

## 3. Results

### 3.1. Study Population and Group Homogeneity

Eventually, and due to school and conservatory dropouts, the study sample comprised 34 musicians (Table 6) and 17 non-musicians; 22 of them were female and 29 of them were male, with an average age of 14 ± 1.7 (Table 7).

In general, both groups were homogenous regarding the evaluated parameters. Only the CPITN assessment regarding the first sextant was significant (*p* = 0.042), as it showed that musicians showed a better CPITN state in the first sextant at the beginning of this study.

### 3.2. Descriptive Statistics and Intraobserver Error

According to the Löe and Silness index, the removal of plaque was 47.1% “good” and just 5.9% “bad” during the review of the first exploration. The same happened in the CPITN, where a 0 value predominated in all sextants, except the fifth sextant, where a 2 value predominated.

The most frequent overjet was of 2–3 mm (Table 8); for the overbite value, 1–3 mm (45.1%) predominated, followed by 2–3 mm (33.3%). Similarly, Angle’s Class I was the most frequent (74.5%). Furthermore, the average crowding and top spacing were 0.55 mm and 0.78mm, respectively. On the other hand, at the lower arch, it was 2.16 and 0.26, respectively (Table 8).

Lower lip erosion affected 21.6% of the individuals, while just 41.2% of them showed erosion facets in the incisors. None of the individuals in this study showed any signs of tooth mobility or deviation, although 11.8% of them did show tooth sensitivity. The presence of labial herpes was observed in 11.8% of individuals, and 37.3% of them showed a lower functional angle to the right. Lastly, just 3.9% of the individuals showed articular noises and none of them showed any signs of bruxism and/or pain muscle. 

In general, both groups were homogenous regarding the evaluated parameters. Only the CPITN assessment regarding the first sextant was significant (*p* = 0.042), as it showed that musicians showed a better CPITN state in the first sextant at the beginning of this study.

While assessing the intraobserver error, it was observed that the measurements were reproducible, except for the dental facets, which showed moderate consistency (Table 9).

### 3.3. Effect of the Instrumental Practice over Time in Different Muscular and Occlusal Levels

This section focuses on the different dental variables evaluated throughout the entire study.

**Figure 2:**
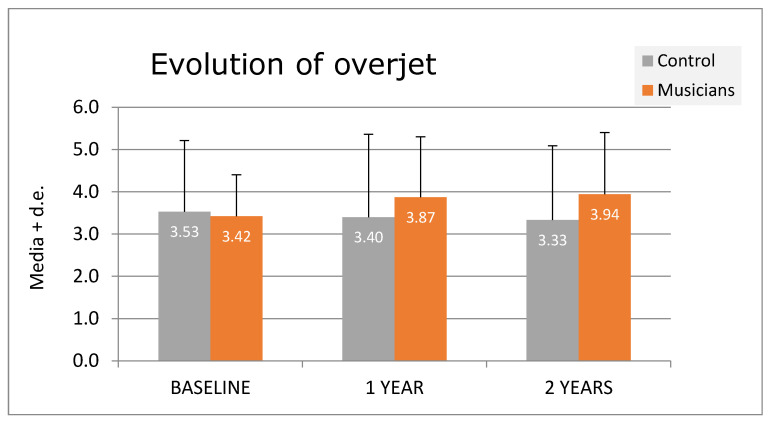
Evolution of overjet over time.

Significant differences were observed regarding overcrowded upper teeth in T2 (*p* = 0,002), overcrowded lower teeth in T2 (*p* < 0.001), overjet in T1 (*p* = 0.019) (Figure 2) and T2 (*p* < 0.001) (Figure 2), lower lip erosion in T1 (*p* = 0.001), and dental erosion in T2 (*p* < 0.001).

Musicians showed higher overjet (Figure 2) and a gradual increase in overcrowding teeth from T0 to T2.

**Figure 3:**
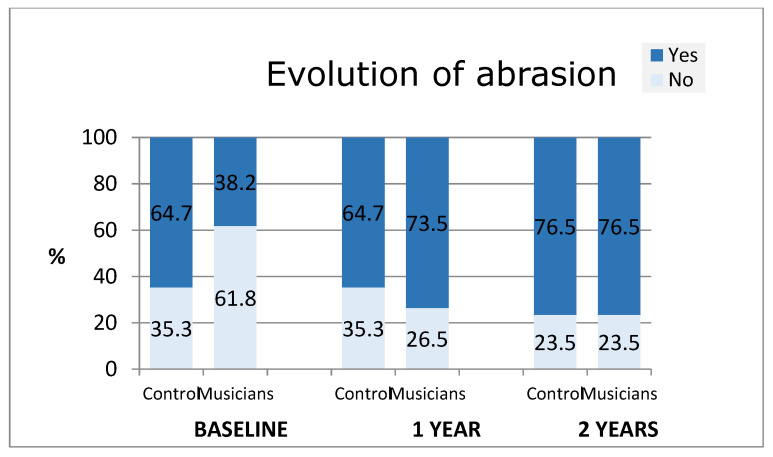
Evolution of enamel abrasion.

Finally, and considering that the prior abrasion condition was worse in the control group, musicians showed a more negative evolution after two years (Figure 3). 

**Figure 4:**
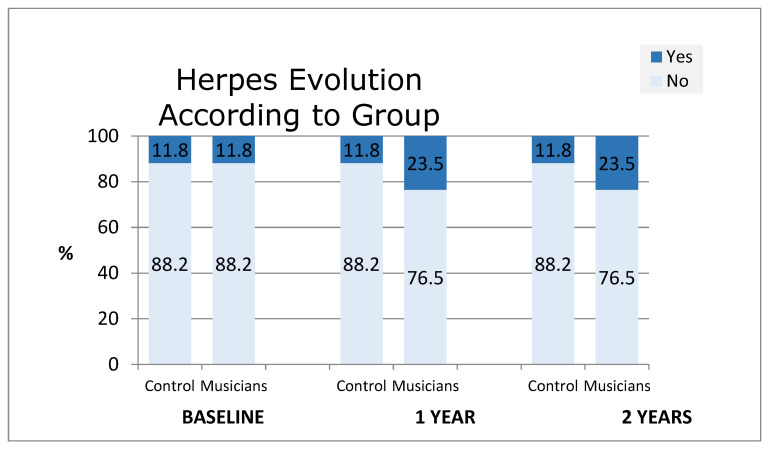
Herpes evolution according to group.

Regarding herpes, no significant differences were found, but there was a tendency towards interaction (Figure 4).

### 3.4. Effect According to the Type of Mouthpiece

**Figure 5:**
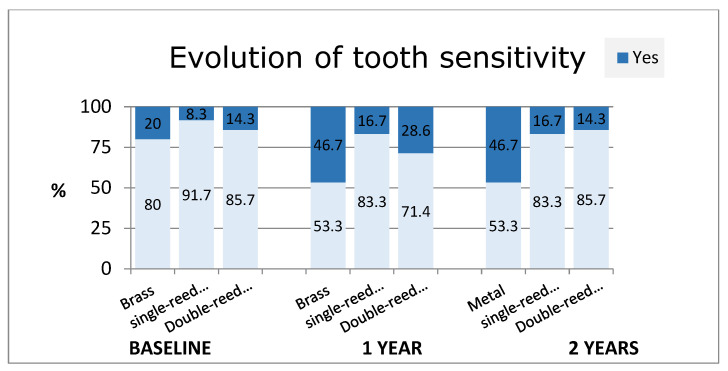
Evolution of tooth sensitivity.

Significant differences regarding tooth sensitivity (*p* = 0.045) were observed, as its increase after 2 years was particularly strong for the group of individuals who used a brass mouthpiece compared to the other two types of mouthpieces, which did not seem strong (Figure 5).

### 3.5. Comparison Between Oral State and Hygiene

**Figure 6:**
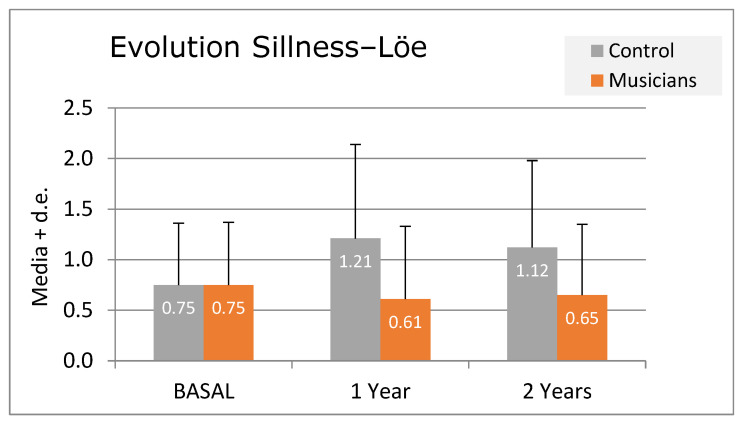
Evolution Silness–Löe.

Significant differences could be seen regarding the index of Löe and Silness in T1 (*p* = 0.002) and T2 (0.012) (Figure 6), and in the CPITN of the first, second, third, fourth, and sixth sextant in T1 (*p*-values 0.004; 0.032; 0.010; 0.011; 0.010; respectively). Those values remain approximately equal in T2.

It was also observed that musicians showed increasing oral health, in contrast with the control group. 

## 4. Discussion

A prospective cohort study was conducted, which is very convenient when conducting a causality study. The music conservatories’ population sample of Torrent, Lliria, Iturbi, and Velluters comprised 30 wind students studying their freshman year at each conservatory. We had access to 34 musicians in total. 

The above-mentioned data highlight the difficulty in obtaining a sample from conservatories. To conduct the explorations, it was first necessary to obtain consent from the conservatory, which is not always easy. Afterward, parental consent was also mandatory. To have access to a wider sample, it would be necessary to count on some institutional support that, in this case, we did not ask for. 

Another limitation of this study is the relatively short observation period of the sample, which was reduced to two years. A longer period to follow the sample would be needed to better assess how instrumental practice affects individuals and to obtain more reliable results. Some variables are indeed significant in this cross-sectional study, which we also previously conducted on advanced students [2]. 

To be able to analyze the first of the goals of this research work, two sets of factors were assessed in this study: on the one hand, factors directly associated with instrumental practice, such as overjet, overbite, lip erosion, Angle Class, overcrowding of teeth, tooth spacing, incisor wear, labial herpes, tooth sensitivity, and devitalization of upper incisors and tooth mobility, and, on the other hand, factors indirectly associated with instrumental practice, such as articular noises, bruxism, and muscular pain.

With regard to factors directly associated with instrumental practice, there were no significant differences regarding overjet in either study group at the first exploration stage, but they significantly increased in the musician group compared to the non-musician group in the two revision phases that followed (*p* < 0.001). This result contrasts with the results shown by Rindisbacher [32] and Grammatopoulos [19], who did not find any significant differences. On the contrary, other authors observed a decrease in overjet in children who played brass instruments, double-reed woodwind instruments, and woodwind head joint instruments, together with an increase in overjet in children who played single-reed woodwind instruments [10,12,13,28]. This may be due to the existing relationship between the mouthpiece and the overjet degree, that is, by increasing or decreasing it. However, in our study, we did not find any significant differences among the various types of mouthpieces. 

Second, no relationship was found between overbite and instrumental practice, which is in accordance with the results obtained by Pang [11] and Grammatopoulos [19]. On the contrary, other studies have found a decrease in overbite in brass instrumentalists, as well as an increase in overbite in double-reed, single-reed, and head joint instrumentalists [12].

Third, labial erosion and/or abrasion affected 21.6% of the total sample of individuals during the first stage, but after 1 and 2 years of instrument practice, it was quite frequent in musicians (61–67%) and practically inexistent in the control group. Labial erosion and/or abrasion most often affect the lower lip or both. These results agree with previous studies in which sores in the lower lip were found in wind instrumentalists [24,25,26].

Fourthly, regarding Angle’s Malocclusion, no significant differences were found between musicians and non-musicians, although we could not prove the previous recommendations made for each kind of mouthpiece according to Angle’s Malocclusion [1,3]. 

Fifth, the results show a higher incidence of tooth overcrowding in musicians over time, particularly in the lower part of the teeth, after two years (*p* < 0.001). This is because playing wind instruments has a small influence on the position of teeth [33]—from tooth mobility in trumpeters [34,35] to lower but most frequent overcrowding of teeth in clarinetists [13]. However, other studies did not obtain any significant differences regarding overcrowding of teeth when playing an instrument [19,32].

Sixth, tooth mobility was evaluated on the grounds of the reasons already explained in the Introduction of this text. The evaluation showed no significant statistical data, although it has not been proven in other articles either, as nobody has previously studied it. 

Seventh, after two years of monitoring, it was observed that incisor wear was higher in musicians. Tooth wear increased in the upper incisors for those playing single-reed woodwind instruments and brass instruments, and in all incisors for those playing double-reed woodwind instruments. These results match Alex’s results [14], which showed that 60% of the assessed musicians (176 musicians) showed dental abrasion. 

Eighthly, no significant differences were observed regarding lip herpes, although there was a tendency towards interaction between herpes and individuals (*p* = 0.098). Musicians tend to develop herpes more, particularly double-reed and head joint brass and woodwind musicians. That same fact was detected by Barkvoll [27] when he studied 45 military musicians. His study showed twice the incidence of herpes in wind instrumentalists than in the control group and non-wind instrumentalists. 

Ninth and last, no statistical significance was achieved, although it was indeed observed that there was a higher incidence of sensitivity in students of music, in agreement with other studies, in which wind instrumentalists showed pulp sensitivity [26,31,35]. On the contrary, no upper incisor devitalization was observed in any of the evaluated incisors. Just one of the musicians suffered from it during the first stage of this study and after two years of monitoring, but it was possibly due to the occlusal trauma he showed due to deep overbite. 

On the other hand, regarding the risk factors indirectly associated with instrumental practice, the results showed, in general, small incidences of articular noises in the sample, together with no significant differences between musicians and the control group. The same happened when evaluating bruxism, which showed no differences between study groups. On the contrary, previous studies have shown higher incidences of articular noises in wind instrumentalists [26,29].

However, some differences regarding muscular pain were indeed found in both groups regarding masseter muscles, orbicularis muscles of the lips, and sternocleidomastoid and zygomatic muscles. The differences showed that musicians were more affected by these muscular pains than those in the control group. Nonetheless, the differences were not statistically significant. These results match those obtained by Sayegh [26], who observed that 33% of musicians showed fatigue in their lip muscles. Even so, if the study was conducted for a longer period, it would be observed, over time, that instrumental practice affects orbicularis and buccinators muscles, as has already been observed in previous studies (*p* = 0.003 and *p* = 0.002, respectively), matching the results obtained by Herman [30]. 

Furthermore, while assessing the second of the objectives of this study, no significant differences were observed among the various types of mouthpieces, possibly due to the small size of the sub-samples studied, except tooth sensitivity, which was indeed higher in brass instrumentalists (*p* = 0.045), given the high forces they produce (500 gr). However, and despite not reaching significant levels (*p* = 0.110), the Silness and Löe index showed a tendency of improvement regarding brass instruments and a worsening regarding double-reed and head joint woodwind instruments from the first stage on. The same happened in CPITN, in which there were certain non-significant tendencies in the first, second, and third sextant regarding better periodontal health in brass and single-reed woodwind instrumentalists. On the contrary, Herman [10] observed higher plaque accumulation in single-reed woodwind instrumentalists. 

Lastly, and while assessing the third of the objectives in this study, that is, hygiene and oral health, it was observed that the control group showed worse hygiene and oral health than musicians, matching the results obtained by Stamatakis [9], who observed an improvement in periodontal health in adult Swiss musicians over the last decades [9], although other studies did observe bad periodontal health in musicians [2,8,25].

On the other hand, if the limitations of this study are analyzed, the first limitation is the sample size. A prospective cohort study was conducted, which is very convenient when conducting a causality study. The music conservatory population sample of Torrent, Lliria, Iturbi, and Velluters comprised 30 wind students studying their freshman year at each conservatory. We only had access to 34 musicians in total. 

To have access to a wider sample, it would be necessary to count on institutional support that, in this case, we did not ask for. 

Because of the foregoing, we suggest continuing with research studies regarding this issue by evaluating different factors. On the one hand, the relationship between mouthpieces of different wind instruments and malocclusions could be evaluated by conducting a longitudinal cephalometric study in order to evaluate the possible changes. 

On the other hand, a mouth guard adapted to each patient and instrument may also be studied and developed, to protect teeth against abrasion, devitalization, and/or tooth mobility, without affecting musical performance. 

Lastly, and as a most innovative research line, the relationship between wind instruments and obstructive sleep apnea syndrome may be studied, given that it is been observed that training of the airway musculature makes it stronger, therefore changing neuronal breathing control plasticity. This may occur during instrumental practice, particularly regarding double-red instruments [36].

## 5. Conclusions

In conclusion, instrumental practice is a risk factor in the development of certain dental conditions, such as greater crowding of teeth, overjet, lip erosion, and dental abrasion in incisors. However, there are no differences between the different existing mouthpieces; only dental sensitivity was greater in brass musicians.

## Figures and Tables

**Table 1:** Exclusion and inclusion criteria.

Inclusion Criteria	Exclusion Criteria
Musician	Played another instrument previously
Wind instrumentalist	
Student of first year professional degree	
Student of Conservatoire of Valencia	

**Table 2:** Degrees of the Silness–Löe index.

Degrees	Interpretation
Degree 0:	No plaque.
Degree 1:	A thin coat of plaque in the gingival margin, just identifiable upon passing the probe.
Degree 2:	A moderate amount of plaque along the gingival margin, free interdental spaces, identifiable at first sight.
Degree 3:	A huge amount of plaque along the gingival margin: interdental spaces are full of plaque.

**Table 3:** Result, interpretation, and clarification of SLI.

Result	Interpretation	Qualification
0.0–0.6	Up to 20% of surfaces of the teeth with bacterial plaque.	“Good”
0.7–1.8	From 21% to 60% of surfaces of the teeth with bacterial plaque.	“Ordinary”
1.9–3.0	From 61% up to 100% of surfaces of the teeth with bacterial plaque.	“Bad”

**Table 4:** Code and needs of treatment according to CPITN value.

Qualification of Sextant	Interpretation	Treatment
0	Periodontal health; absence of bleeding	No need for treatment
1	Bleeding on correct probing	Dental prophylaxis and brush-up training
2	The presence of calculus and/or overhangs	Treatment of 1 + professional tooth rasping
3	Presence of 4 and/or 5 mm deep pathological pockets (partially visible black stripe of the probe)	Treatment of 1 + professional tooth rasping
4	Presence of 6 mm or more, deep pathological pockets (no visible black stripe)	Treatment of 3 + periodontal surgery

**Table 5:** Degrees of dental abrasion.

Code	Condition
0:	Surface/facets
1:	Enamel
2:	Enamel with dentin of up to 1 mm
3:	The dentin of more than 1 mm
4:	Up to 1/3 dental crown
5:	More than 1/3 dental crown

**Table 6:** Summary of the sample studied.

Family of Instruments	No.	Instrument	%
Single-reed woodwind	12	Clarinet	26.5
		Saxophone	8.8
Double-reed and head joint woodwind	7	Oboe	8.8
		Flute	11.8
Brass	15	Trumpet	14.7
		French horn	20.6
		Trombone	5.9
		Saxhorn	2.9
Total	34		100

**Table 7:** Summary of population sample: age and sex.

		Group		
		Experimental	Control	Total
Total	N.	34	17	51
	%	100	100	100
Women	N.	16	6	22
	%	47.1	35.3	43.1
Men	N.	18	11	29
	%	52.9	64.7	56.9
Age	Median	14.1	13.7	14.0
	Standard deviation	2.0	1.0	1.7
	Minimum	11.0	13.0	11.0
	Maximum	20.0	16.0	20.0

**Table 8:** Results of the descriptive statistics.

Variable	Minimum Value (mm)	Maximum Value (mm)	Median (mm)
Overjet	0	6	3.53 ± 1.23
Upper overcrowding	0	3.5	0.55 ± 0.87
Lower overcrowding	0	6.5	2.16 ± 1.61
Upper teeth spacing	0	6	0.78 ± 1.28
Lower teeth spacing	0	3.5	0.26 ± 0.85

**Table 9:** Results of intraobserver error evaluation.

	D Dahlberg	I. Kappa	% Matching over n = 10
Overjet	0.00		
Overbite	0.00		
Upper overcrowding	0.16		
Lower overcrowding	0.19		
Upper teeth spacing	0.00		
Lower teeth spacing	0.19		
Angle Class		0.71	90%
Dental facets		1.00	100%
Dental abrasion		0.64	80%
Enamel up to 1 mm		1.00	100%

## Data Availability

The original contributions presented in the study are included in the article, further inquiries can be directed to the corresponding author.
